# CrisprBuildr: an open-source application for CRISPR-mediated genome engineering in *Drosophila melanogaster*

**DOI:** 10.1093/g3journal/jkaf251

**Published:** 2025-10-21

**Authors:** Nicole Horsley, Adam von Barnau Sythoff, Mark Delgado, Selina Liu, Clemens Cabernard

**Affiliations:** Department of Biology, University of Washington, Life Science Building, Seattle WA 98195, United States; Department of Biology, University of Washington, Life Science Building, Seattle WA 98195, United States; Department of Biology, University of Washington, Life Science Building, Seattle WA 98195, United States; Department of Biology, University of Washington, Life Science Building, Seattle WA 98195, United States

**Keywords:** CRISPR, *Drosophila*, genome engineering

## Abstract

CRISPR/Cas9 is a powerful tool for targeted genome editing experiments. Using CRISPR/Cas9, genes can be deleted or modified by inserting specific DNA sequences, encoding for fluorescent proteins, small peptide tags, or other modifications. Such experiments are essential for detailed gene and protein characterization. However, designing and cloning the corresponding constructs can be repetitive, time-consuming, and laborious. To assist users in CRISPR/Cas9-based genome engineering, we developed CrisprBuildr, an open-source, web-based application for designing modifications to their target genes. CrisprBuildr guides users through creating guide RNAs and repair template vectors to generate cloning maps. The application is designed for the *Drosophila melanogaster* genome but can serve as a template for other available genomes. We also created new tagging vectors using EGFP and mCherry combined with the small peptide SspB-Q73R for use in iLID-based optogenetic experiments.

## Introduction

Genome engineering, such as deleting genes, introducing point mutations, or inserting defined DNA sequences at specific loci, has been revolutionized with the advent of CRISPR/Cas9 (Sternberg et al. 2014). The ability to precisely manipulate the genome has significantly enhanced the molecular genetic toolkit from bacteria to human cells. CRISPR/Cas9 also advances basic research with classic model organisms such *Drosophila melanogaster*. ∼ 65% of human disease genes are conserved in *Drosophila* (Baldridge et al. 2021), and loss-of-function and protein localization studies aid in the functional characterization of genes and molecular processes.

Visualizing protein localization with high spatial and temporal resolution is a key tool to understand protein function. Fusing proteins of interest with fluorescent proteins (FP) for live cell analysis has become a powerful tool to study protein function in various cellular contexts. Of particular interest are genetically encoded FP fusions, since they do not need to be transfected for each experiment (Kanca et al. 2017). Traditionally, genetically encoded fusion proteins were inserted in random locations in the genome. Their expression could either be controlled via endogenous promoters or enhancers, usually cloned upstream of the fusion protein, or through binary expression systems. For instance, the Gal4/UAS system has been used extensively to express FP fusions under the control of tissue-specific Gal4 lines in *Drosophila melanogaster* (Brand and Perrimon 1993) as well as zebrafish (Scheer and Campos-Ortega 1999).

With the discovery of CRISPR/Cas9-based genome engineering methods, it is now possible to integrate FPs with high precision, replacing unlabeled proteins with genetically encoded protein-FP fusions (Bier et al. 2018). CRISPR/Cas9 creates double-stranded breaks (DSBs) at defined positions in the fly genome by guiding the endonuclease Cas9 with a programmable guide RNA (gRNA) (Gratz et al. 2013a, 2013b; Gratz et al. 2014; Port et al. 2014; Port and Bullock 2016; Kanca et al. 2019, 2022; Gratz et al. 2023a; Gratz et al. 2023b; Aguilar, et al. 2024b). Such DSBs can either be repaired using nonhomologous end joining (NHEJ) or homology-directed repair (HDR) pathways. For the purpose of endogenous gene tagging, the HDR pathway is preferred, which requires single or double-stranded exogenous DNA as repair templates (Bier et al. 2018). Designing CRISPR-based genome engineering experiments can be performed routinely in labs with basic experience in molecular biology, and step-by-step protocols and web resources have been published previously (Bier et al. 2018; Gratz et al. 2023a; Aguilar, et al. 2024a). However, planning and designing constructs for CRISPR experiments can be challenging, time-consuming, and laborious. Here, we report the generation of a new set of vectors and a web-based cloning tool, called CrisprBuildr, to aid in the planning and cloning of repair plasmids for generating deletion alleles or for tagging proteins of interest at the endogenous locus with FPs. CrisprBuilder is a bricolage of existing web tools, assembled into a step-by-step workflow, guiding users through the deletion or tagging process in silico. CrisprBuildr reduces the time needed to plan and design CRISPR-based genome engineering experiments. The tool also provides a platform to train new users on CRISPR-based genome engineering experiments. CrisprBuilder is built on an open platform, and we envision it to be extended by including other tagging vectors, genomes, or CRISPR-based applications.

## Materials and methods

### Fly strains and genetics

The following fly strains were generated in this study: *SspB::EGFP::aPKC, SspB::EGFP::AurB, AurB::EGFP::SspB, Sqh::EGFP::SspB*. Transformants were delivered and screened for eye DsRed expression. The DsRed cassette was floxed out by crossing to hsCre.

### Plasmid cloning

To generate vectors for *N*-terminal tagging, EGFP or mCherry (FP, hereafter) was first cloned into pHD-SspB-R73Q-DsRed utilizing restriction sites NdeI and Pmel to generate pHD-SspB-R73Q::FP-DsRed. An Mlul restriction site was inserted between the SspB and the FP to allow for easy swapping of the fluorophore for future applications. *N*-terminal homology arms corresponding to a gene of interest can be cloned into this backbone using restriction sites EcoR1 and NotI or NdeI. C-terminal homology arms can be inserted into AscI and Xhol for the C-terminal homology arm.

To generate C-terminally tagged vectors EGFP or mCherry (FP, hereafter) was inserted into pHD-SspB-R73Q-DsRed utilizing restriction sites Not1 and Mlul to generate pHD-FP::SspB-R73Q-DsRed, conserving both restriction sites for future fluorophore swapping. *N*-terminal homology arms corresponding to a gene of interest can be cloned into this backbone using restriction sites Srf1, EcoR1, and NotI. C-terminal homology arms can be inserted between and BgIII and XhoI. All cloning steps were performed using InFusion cloning from Takara.

Both *N*- and C-terminal tagging vectors contain an extended flexible linker between SspB-R73Q and the FP.

The following final vectors were made:

*For N-terminal tagging:*

pHD-SspB-ExLK-EGFP-DsRed

pHD-SspB-ExLK-mCherry-DsRed

*For C-terminal tagging:*

pHD-EGFP-ExLK-SspB-DsRed

pHD-mCherry-ExLK-SspB-DsRed

### Live cell imaging

72 to 96 h after egg laying, larval brains were dissected using microdissection scissors (Fine Science Tools catalog number 15003-08) (Segura and Cabernard 2023) and forceps (Dumont #5, Electron Microscopy Sciences, item number 0103-5-PO) in Schneider's medium supplemented with 10% bovine growth serum (HyClone, item number SH30541.03) and transferred to chambered slides (Ibidi, catalog number 80826) for imaging. Live samples were imaged with an Intelligent Imaging Innovations (3i) spinning disc confocal system, consisting of a Yokogawa CSU-W1 spinning disc unit and 2 Prime 95B Scientific CMOS cameras. A 60×/1.4NA oil immersion objective mounted on both microscopes was used for imaging. Live imaging voxels are 0.22 × 0.22 × 1 μm (60×/1.4NA spinning disc).

### CrisprBuildr

CrisprBuildr (http://crisprbuildr.org) connects to www.flybase.org and cross-references the user’s search term with available data from Flybase. If a relevant match is found, it will ping the flybase api in order to pull the raw genetic data. Targets around either the start or stop codon are retrieved from http://targetfinder.flycrispr.neuro.brown.edu/. Target efficiency is checked with www.flyrnai.org/evaluateCrispr/. The application will then look for primers within 400 to 600 bases and 1000 to 1200 bases, both upstream and downstream from our target area. It will submit this information via simulated browser to bioinfo.ut.ee/primer3-0.4.0/ and will return the selection to the user. Once the user finishes selecting their primers, the application will pull the relevant Oligo information from http://targetfinder.flycrispr.neuro.brown.edu/.

The source code and a detailed application description can be found here: https://github.com/CabernardLab/CrisprBuildr.git

## Results and discussion

### Construction of new protein tagging vectors for protein localization and mislocalization experiments

**Fig. 1.**
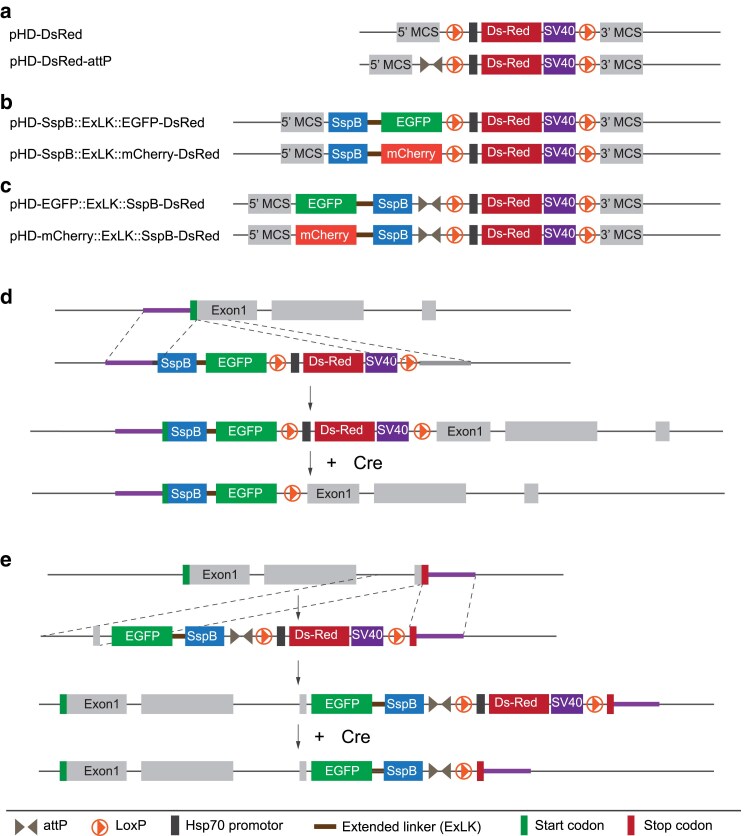
Tagging vectors for Crispr/Cas9 genome engineering. a) Existing vectors used for creating gene deletions. New vectors for b) N-terminal or c) C-terminal tagging experiments. The vectors allow for the insertion of SspB::EGFP or SspB::mCherry cassettes for iLID-mediated optogenetic experiments and protein localization studies. d) After inserting the *SspB::EGFP/mCherry* at the N-terminus or e) the *mCherry/EGFP::SspB* at the C-terminus, the *DsRed* cassette can be floxed out using the existing LoxP sites.

We constructed a new set of cloning vectors intending to (i) tag genes of interest at their endogenous locus for localization studies and (ii) combine it with the optogenetic iLID (Guntas et al. 2015) system for protein mislocalization experiments. To this end, we modified the previously published *pHD-DsRed-attP* vector (Gratz et al. 2014) (Fig. 1a), inserting a cassette containing *SspB(R73Q)* and the cDNA encoding for a FP (*EGFP or mCherry*) upstream of *DsRed* for either *N* or C-terminal tagging. SspB(R73Q) is a 13kD adaptor protein from *E. coli* with improved binding affinity to the *E. coli* protein SsrA (Guntas et al. 2015). *pHD-SspB::FP-DsRed, pHD-FP::SspB-DsRed* vectors generate fusion proteins compatible with the optogenetic iLID system (Guntas et al. 2015), which utilizes 7 residues of SsrA, inserted into the photoactive LOV2 element of Avena sativa (AsLOV). *SspB* is cloned in frame with FPs and either located at the very *N*- or C-terminus to be accessible for iLID's SsrA residue (Fig. 1b, c). We previously demonstrated the utility of the iLID system *in vivo* by trapping centrosomal proteins on centrioles in dividing fly neuroblasts (Gallaud et al. 2020). We retained the 2 *LoxP* sites of *pHD-DsRed* and used the remaining *LoxP* site as a peptide linker between the protein of interest and the SspB-FP cassette after Cre-mediated site-specific recombination (Fig. 1d, e). The 2 multiple cloning sites are used to clone in the homology arms for the desired locus.

To demonstrate the utility of these vectors, we inserted the SspB::EGFP or EGFP::SspB into the *N*-terminus or C-terminus of the Par complex component atypical Protein Kinase C (aPKC; Protein Kinase Cι in vertebrates), AuroraB (AurB; AurK in vertebrates) and Myosin's regulatory light chain Spaghetti Squash (Sqh; MYL9 in vertebrates). To this end, we cloned in homology arms of the corresponding genes into the modified *pHD-SspB:::EGFP-DsRed-attP* or *pHD-EGFP::SspB-DsRed-attP* vector and injected the plasmids together with guide RNAs into Cas9 expressing embryos. After floxing out the DsRed cassette of successful knock-in lines, we imaged third instar larval neuroblasts expressing these fusion proteins together with the microtubule marker mCherry::Jupiter (Karpova et al. 2006; Cabernard and Doe 2009).

**Fig. 2.**
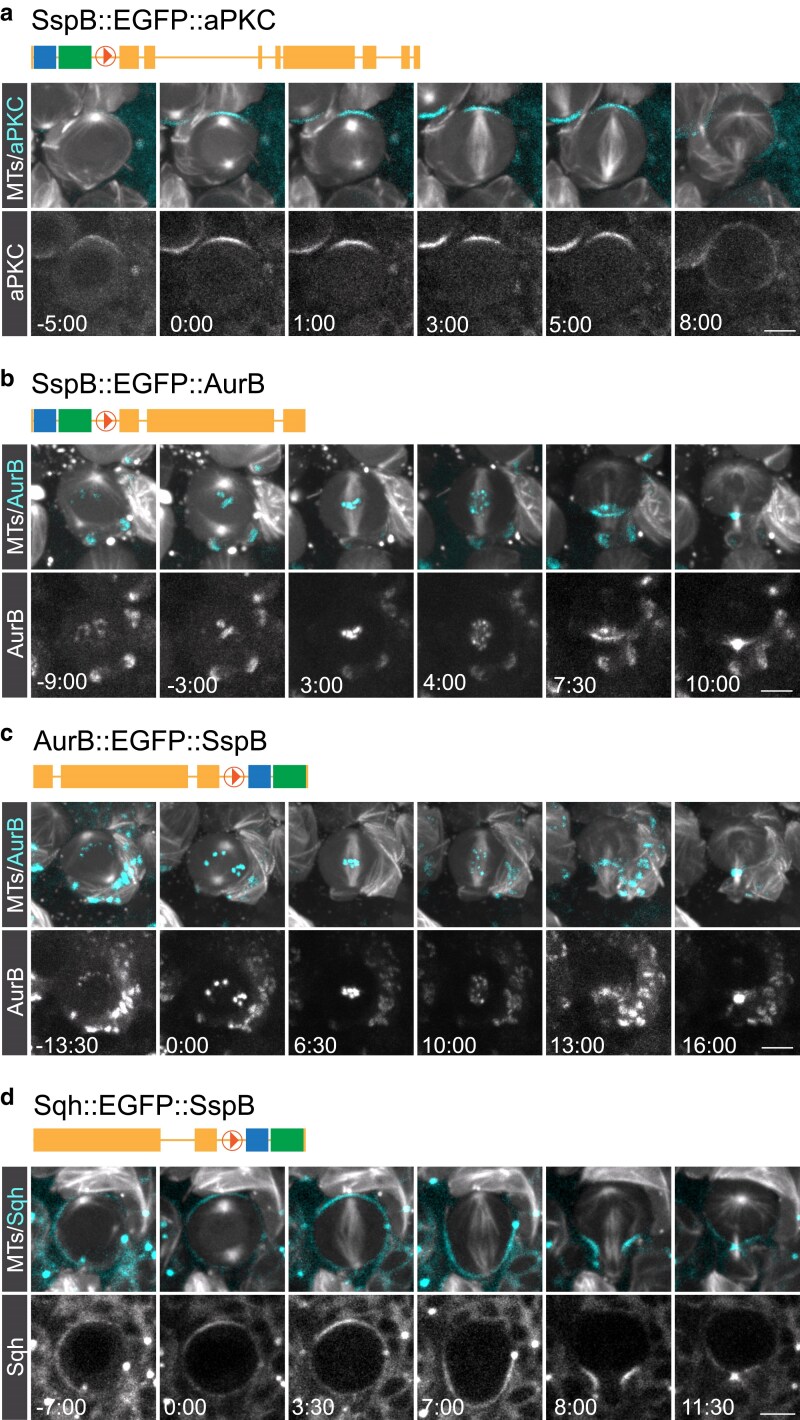
Example fly lines expressing fusion proteins in fly neuroblasts. a) N-terminal tagging of aPKC with SspB::EGFP. aPKC correctly localizes on the apical neuroblast cortex throughout mitosis. b) N-terminal and c) C-terminal tagging of AuroraB with SspB::EGFP and EGFP::SspB, respectively. Both lines show AurB localized on kinetochores and the cleavage furrow in telophase. d) C-terminal tagging of the Myosin's regulatory subunit Spaghetti Squash (Sqh) with EGFP::SspB. Sqh::EGFP correctly localizes at the neuroblast cortex throughout mitosis. Scale bar is 5 μm; Time in min:sec.

In mitotic fly neuroblasts, aPKC is localized on the apical neuroblast cortex (Wodarz et al. 2000; Gallaud et al. 2017), which is accurately reproduced by our *SspB::EGFP::aPKC* line (Fig. 2a). AurB is a member of the chromosome passenger complex (CPC) and both SspB::EGFP::AurB and AurB::EGFP::SspB were correctly localized to the centromeric region as well as the ingressing cleavage furrow, similar to other CPC components (Roth et al. 2015) (Fig. 2b, c). Finally, Sqh::EGFP::SspB is transitioning from a cortical localization, with transient apical enrichment, to the cleavage furrow as previously described with other Sqh::EGFP transgenic lines (Royou et al. 2002; Barros et al. 2003; Cabernard et al. 2010; Roubinet et al. 2017; Tsankova et al. 2017) (Fig. 2d). We conclude that the insertion of the *SspB::EGFP* or *EGFP::SspB* at the endogenous locus results in fusion proteins with accurate localization dynamics in fly neural stem cells.

### CrisprBuildr, an open-source, customizable web application for designing gene deletion and tagging constructs

**Fig. 3.**
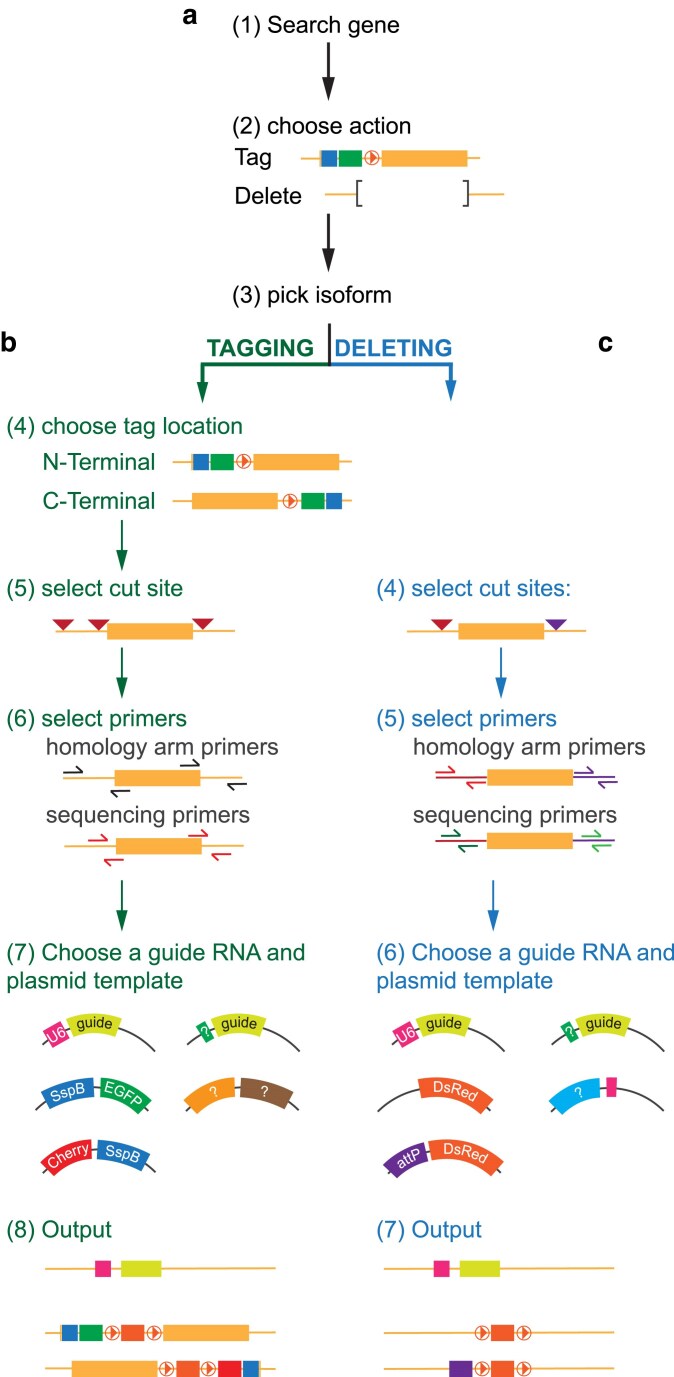
CrisprBuildr decision tree. CrisprBuildr, an open-source genome engineering application, allows users to design deletion and tagging experiments *in silico*. a) After entering a *Drosophila* gene of interest, users can b) tag or c) delete genes of interest. The application prompts users to pick the desired gene isoform, suggests Cas9 cutting sites, and proposes primers to amplify and sequence homology arms. Users can pick from a selection of guide RNA, tagging, or deletion vectors, including their own. The application output are (1) *a pU6-BbsI-chiRNA* or custom guide RNA vector map containing the selected guide RNAs, (2) an unaltered map of the genomic locus, and (3) a map containing the selected cassette in the desired location: *SspB::EGFP/mCherry* for N-terminal tagging, *mCherry/EGFP::SspB* for C-terminal tagging or a custom cassette for N-, C-terminal tagging. For deletion experiments, the application provides the *attP-DsRed* and *DsRed* vectors, respectively. As before, users can upload their own deletion vectors to incorporate homology arms.

Next, we sought to streamline the generation of CRISPR-based genome engineering experiments. To this end, we built a custom-made cloning app, called CrisprBuildr (http://crisprbuildr.org). CrisprBuildr aids users in the design of (i) deletion and (ii) tagging experiments for genes of their choice. Specifically, CrisprBuildr allows users to generate cloning maps for deletion or tagging experiments. CrisprBuildr accepts Flybase gene symbols or Flybase gene IDs. CrisprBuildr connects to Flybase (www.flybase.org) to retrieve the locus information of the gene of interest. Users can choose to design deletion or tagging experiments after inserting the gene of interest in the search field. In both cases, the desired isoform can be selected from a pull-down list. Since CrisprBuildr is currently not designed as a full-fledged genome browser, we strongly encourage users to first consult Flybase (www.flybase.org) to pick the gene isoform best suited for their experiment. Furthermore, we strongly recommend that users first analyze the gene region of the targeted gene and isoform to avoid changing regulatory elements, which could negatively impact gene expression. For tagging experiments, users are prompted to select *N*- vs C-terminal tagging, and based on this decision, CrisprBuildr will search around the start or stop codon for suitable target sites to induce DBSs. To this end, CrisprBuildr is interacting with http://targetfinder.flycrispr.neuro.brown.edu/ to find suitable CRISPR sites and subsequently cross-checks the sites’ efficiency by connecting to www.flyrnai.org/evaluateCrispr/. After selecting the best CRISPR cut sites, CrisprBuilder will search for suitable primer sites within 1000–1200 and 400–600 bases, both upstream and downstream from the target area, to search for suitable amplification and sequencing primers, respectively. These primers are used to PCR amplify and sequence the genomic region around the target site to identify polymorphisms and other genetic alterations that could interfere with the experiment. Users might design their own final cloning primers based on their respective needs. Since homologous recombination is dependent on the size of the inserted cassette (Beumer et al. 2008), we aimed for ∼1000 bp homology arms (see Fig. 1d, e) flanking the desired insertion site for our tagging vectors. However, shorter integration cassettes can be efficiently inserted using shorter homology arms (Kanca et al. 2019, 2022) (see also below). CrisprBuildr provides amplification and sequencing primers to amplify and sequence prospective homology arms. It will submit this information via simulated browser to bioinfo.ut.ee/primer3-0.4.0/ and will return the selection to the user. As a last step, users can either choose a *pHD-SspB::FP-DsRed* (for *N*-terminal tagging) or *pHD-FP::SspB-DsRed* (for C-terminal tagging), containing either an EGFP (Cormack et al. 1996) or mCherry fluorescent tag (Shaner et al. 2004). EGFP and mCherry are some of the most used FPs in tagging experiments and are compatible with most fluorescent microscope excitation and emission optics. It is noteworthy that while some reports suggest mCherry fusion proteins form aggregates (Landgraf et al. 2012), others report that mCherry oligomerization occurs only weakly (Costantini et al. 2015 ). To provide additional flexibility in designing tagging experiments, users can also upload their own vector with designated spaces to insert homology arms (Fig. 3a, b).

CRISPR-based mutagenesis experiments can be achieved by generating 1 or 2 DSBs using 1 or 2 guide RNAs. Single and double DSBs can be repaired via NHEJ, creating small insertions and deletions (indels). However, indel generation is not targeted, forcing users to screen for the most deleterious events. Furthermore, NHEJ does not permit the introduction of a dominant marker that can be used to screen for targeting events. Alternatively, targeted deletions or point mutations can be created by creating DSBs, which are then repaired via HDR. To that end, a repair template needs to be provided that contains homology arms for the targeted region and a dominant marker used to screen for successful targeting events. HDR-mediated genome engineering can be induced with either 1 or 2 DSBs, albeit 2 DSBs might increase efficiency. HDR-mediated genome modifications further allow the introduction of targeted point mutations, incorporated into the homology arms. However, in this case, users are still required to use molecular screens to ensure the mutation has been incorporated. To create targeted deletions, users can pick an *N*’ and C’ terminal cutting site in CrisprBuildr, a strategy that has been used successfully in original gene deletion experiments (Gratz, et al. 2013a; Gratz et al. 2014; Port et al. 2014). After choosing high-efficiency CRISPR sites with as few off-targets as possible, the application provides amplification and sequencing primers for both the *N*- and C-termini. As before, these primers are used to amplify the region surrounding the *N*- and C-terminus containing the chosen guide RNAs to screen for polymorphisms that might interfere with the targeting event. Users can pick *pHD-DsRed* or *pHD-DsRed-attP* as integration vectors, replacing the targeted gene (Fig. 3c). Alternatively, users can upload their own vector for gene deletion events.

For both operations, the application provides a printable project information review and 3 downloadable cloning maps: (i) a guide RNA vector map containing the selected guide RNAs to pilot the Cas9 endonuclease into their target sites for proper double strand breakage; (ii) a gene locus map without the chosen tag, containing the chosen gene isoforms and primers to amplify the homology arms as well as sequencing primers; (iii) a gene locus map with the *SspB::FP-DsRed, FP::SspB-DsRed, DsRed, DsRed-attP* or user-define cassette, inserted at the desired site. This map also displays the locus with the amplified homology arms. Ideally, this map can be used as a reference map to clone the corresponding homology arms. It is critically important that users also introduce a silent mutation into the PAM site because failure to disrupt the gRNA target site or PAM sequence in the donor will result in recutting of the repaired allele, leading to indels or incomplete edits. We recommend that users consult the helpful protocols and suggestions provided at FlyCrispr (https://flycrispr.org/) for further guidance. While the current version does not allow for automatically mutating the PAM site in the final gene locus map, CrisprBuildr upgrades might include this feature. It is further important that users verify the reading frame after LoxP floxing. Finally, users are strongly encouraged to manually check the gRNA vectors to ensure that the selected target sites indeed occur in the desired target region.

These maps can be downloaded and viewed in Ape (https://jorgensen.biology.utah.edu/wayned/ape/) or cloning applications accepting Genbank file format (.gb). A short CrisprBuilder user manual is linked to the application. The application also includes a link to a bug report form for users who experience problems. Bug reports are monitored and addressed in a timely fashion.

CRISPR-based genome engineering opened the door for targeted genome alterations with unprecedented precision. However, designing the necessary vectors and constructs can be time-intensive and require the necessary expertise. Here, we report the generation of new vectors to tag genes at the endogenous locus with CRISPR in *Drosophila melanogaster*. These vectors allow the insertion of FPs—EGFP or mCherry—combined with the short iLID-binding peptide SsrA at the N- or C-terminus of fly genes of interest. We also built a web-based application, called CrisprBuildr (http://crisprbuildr.org), that aids users in the design of deletion or tagging experiments. The app links to existing databases that identify Cas9 cut sites, aid in primer design, and retrieve gene locus information from existing databases. CrisprBuildr guides users through the process in a stepwise fashion and generates an unaltered map of the locus of interest as well as a map of the locus after the deletion or insertion. This map can provide a blueprint to aid users in the cloning process and can be used for effective teaching in the design and cloning of constructs for CRISPR-Cas9 experiments.

While CrisprBuildr is an application to aid users in planning and designing CRISPR experiments, CRISPR-mediated genome engineering can also induce unwanted alterations. For instance, integration of vector backbone sequences or multiple copies of the donor construct represents common undesired outcomes in knock-in experiments. While this is less of a problem for gene-disruption experiments, gene tagging might be negatively impacted. Users should devise PCR-based strategies, Southern blotting, or 5′ end modifications in long homology arms to favor single copy cassette insertion (Gutierrez-Triana et al. 2018; Skryabin et al. 2020; Luqman et al. 2025).

To prevent unwanted vector backbone integrations, users could also excise the tagging cassette using gRNAs, targeting sequences flanking the insertion cassette to create a linearized DNA template for the HDR of the locus (Lamb et al. 2017; Kanca et al. 2019, 2022; Aguilar, et al. 2024b).

It is also important to note that the tagging vectors *pHD-SspB::FP-DsRed* and *pHD-FP::SspB-DsRed* contain the eye marker *3xP3-DsRed* to screen for successful integration events. *3xP3-DsRed* is flanked by 2 *LoxP* sites, which are floxed out to create an in-frame fusion between the gene of interest and the chosen *SspB::FB/FB::SspB* cassette. However, *LoxP*-mediated site-specific recombination will leave one *LoxP* site behind. We took advantage of this “scar” and use it as a linker between the gene and *FB::SspB* tag. The fusions we created with this strategy generated successful integration with functional fusion proteins (Fig. 2). However, users might want to consider designing “scarless’ tagging experiments. To that end, different strategies have been proposed to remove the scar. For instance, flanking the *3xP3-DsRed* marker cassette by PBac transposon ends allows for the removal of the *3xP3-DsRed* marker by mobilizing the PBac transposon (https://flycrispr.org/scarless-gene-editing/). Alternatively, Aguilar and colleagues inserted 2 gRNAs with no cutting sites in the fly genome outside of the *3xP3-DsRed* marker. After successful integration of the cassette at the right location, *3xP3-DsRed* can be excised to create scarless tagging (Aguilar et al. 2024a, 2024b). With the option to upload a user-defined homology arm vector, CrisprBuildr provides the flexibility to design customizable, scarless gene editing experiments. To deliver the gRNAs, we currently integrated pU6-BbsI-chiRNA in CrisprBuildr, but users can also upload their own template to integrate guides.

CrisprBuildr is ready to use but offers opportunities for future expansion. For instance, for gene-tagging experiments, we routinely mutate the PAM site in the donor construct to avoid Cas9 cutting in the repair template. In its current form, the application does not incorporate an automated way to mutate the PAM site, requiring users to mutate it manually in the final cloning map and to design the cloning strategy accordingly.

Future upgrades in CrisprBuildr could also aid users in generating site-specific point mutations using prime editing (Bosch et al. 2021). Similarly, it will be interesting to expand CrisprBuildr to incorporate other Cas enzymes such as Cas12a, enabling temperature-based control of cutting (Port et al. 2020) or PAM-relaxed Cas9 variants (Ni et al. 2020; Zirin et al. 2022). Currently, CrisprBuildr is only retrieving genomic information for *Drosophila melanogaster*, but since the application is completely open-source, we envision that future versions could incorporate genomes from other *Drosophila* species or other established or emerging model organisms.

## Data Availability

N- and C-terminal tagging plasmids reported here will be available upon request and will be deposited on DGRC. Fly strains and plasmids made for the lines in Fig. 2 are available upon request. CrisprBuildr code is available on GitHub (https://github.com/CabernardLab/CrisprBuildr.git) (Liu 2025). CrisprBuilder is accessible on http://crisprbuildr.org.
